# Reinterventions and medical costs after tetralogy of Fallot repair: a retrospective cohort study using health insurance claims in Japan

**DOI:** 10.1007/s11748-025-02174-7

**Published:** 2025-06-23

**Authors:** Yasutaka Hirata, Shintaro Nemoto, Yusei Hamada, Akihiro Nakajima, Yasumi Nishiwaki, Kosuke Kikuchi

**Affiliations:** 1 Department of Cardiovascular Surgery, National Center for Child Health and Development, 2-10-1 Okura, Setagaya-Ku, Tokyo, 157-8535 Japan; 2 Department of Thoracic and Cardiovascular Surgery, Osaka Medical and Pharmaceutical University, Osaka, Japan; 3 Implantable Medical Device Development Department, Teijin Limited, Tokyo, Japan; 4 Clinical Development Control Department, Teijin Pharma Limited, Tokyo, Japan

**Keywords:** Congenital heart disease, Tetralogy of Fallot, Reintervention, Insurance claims data

## Abstract

**Background:**

Reinterventions after congenital heart disease surgery include not only reoperations but also medical catheter interventions, and the details of these treatment realities are often unclear. This study aimed to elucidate the medical and surgical reinterventions and associated medical costs after the tetralogy of Fallot (TOF) repair using Japanese health insurance claims data.

**Methods and results:**

We analyzed reinterventions and medical costs from insurance claims data of patients who underwent TOF repair between 2005 and 2021. Of 174 patients who underwent TOF repair, 23 (13.2%) received a total of 34 reinterventions. These included 23 percutaneous catheter interventions and 11 reoperations. The 5-year reintervention-free rate was 87.5% overall, 94.9% for surgeries with right ventricular outflow tract reconstruction (N = 130), and 65.6% for surgeries with peripheral pulmonary artery plasty (N = 44). The median (interquartile range) medical cost for patients without reintervention was ¥5.33 million (4.62–7.14 million) and the cost for the patients with reintervention was ¥ 10.59 million (7.73–13.97 million).

**Conclusion:**

Using Japanese insurance claims data, we analyzed the reoperation and catheter intervention after the TOF repair. The reintervention-free rate after TOF repair differed significantly by surgical procedure with a tendency for poorer postoperative prognosis, particularly in cases involving the peripheral pulmonary artery plasty. These analysis results may contribute to predicting outcomes after TOF repair for healthcare professionals.

With advancements in medical technology, many patients with congenital heart disease (CHD) who undergo surgery in childhood now reach adulthood. Detailed analysis of long-term postoperative outcomes and feedback to treatment strategies are increasingly important for improving long-term treatment outcomes and quality of life for patients and their families [1, 2]. In the long-term follow-up after CHD surgery, continuous monitoring by pediatrics and internal medicine is required, and the frequency of reinterventions is high. These reinterventions include not only surgical methods through reoperation but also percutaneous interventions through catheter treatment [3]. However, previous studies on reinterventions have often been limited to a small number of facilities and do not capture nationwide trends. There are few reports on general trends that are cross-sectional across multiple medical departments on a national scale.

Studies targeting a small number of facilities are easier to standardize and manage data collection criteria, allowing for detailed data collection on patients and surgical techniques [4]. However, generalization is difficult. By utilizing nationwide databases, it is possible to increase patient coverage and obtain evidence with high generalizability, which is why database analyses on a national scale are being conducted in various countries worldwide [5, 6].

One of the representative nationwide databases for CHD treatment in Japan is the Japan Cardiovascular Surgery Database (JCVSD), which has been accumulating data since the early 2000s. It regularly reports on mortality rates and frequencies of major CHD surgeries [5]. However, the data collected in this database is limited to surgical interventions during the hospitalization period associated with the surgery, restricting the evaluation of medical interventions and long-term reinterventions.

Japan has a universal health insurance system, and when insurance-covered medical care is provided, medical institutions issue detailed statements of medical expenses. The database that compiles these statement data for each patient is the insurance claims data, which allows for the understanding of diagnoses, materials, and drug prescriptions for both inpatient and outpatient care [7]. Although detailed analysis of medical procedures received by patients is more challenging compared to medical record information, as it is a record for medical fee calculation, it is useful as a tool for evaluating the implementation status of medical procedures performed in the long term, as it is less affected by changes in departments or hospitals [8]. Additionally, as it is comprehensive information on insurance-covered medical care provided to patients, it allows for simultaneous analysis of both surgical and medical interventions. It is also possible to calculate the medical costs incurred for patient treatment from insurance claims data. CHD treatment requires long-term medical care even after surgery, and the associated medical costs become a social economic burden [9]. Therefore, it is important to clarify the disease burden, such as the frequency of reinterventions and medical costs, to consider cost-effective treatment strategies.

In this study, we analyzed the realities of reinterventions and their associated medical costs after surgical treatment for tetralogy of Fallot, a representative CHD, using insurance claims data.

## Patients and methods

### Data source and study population

This study is a retrospective cohort analysis using a nationwide database owned by JMDC Inc. (Tokyo, Japan). JMDC maintains a database of anonymized insurance claims, health checkup, and health insurance subscriber registry data collected from health insurance societies in Japan for epidemiological and public health purposes [10]. From the insurance claims data, information on diagnoses based on ICD-10, prescribed drugs based on ATC classification, and medical procedures can be obtained. In this study, we analyzed insurance claims data of insured individuals with health insurance enrollment records, with a total data period from January 2005 to December 2022.

The enrollment period was from January 2005 to December 2021, and patients who underwent TOF repair during this period were included, with the date of surgery calculation set as the index date. TOF repair was defined as TOF repair (with right ventricular outflow tract reconstruction) (procedure code: 150,146,510) or TOF repair (with peripheral pulmonary artery plasty) (procedure code: 150,146,610). Patients who underwent pulmonary artery banding (procedure code: 150,139,110) until the day before the index date, those who had multiple TOF surgeries, or those who were 18 years or older on the index date were excluded. Follow-up ends at the end of the year in which enrollment in the health insurance society ends or December 2022, whichever is earlier.

Patient background, reinterventions, and medical costs were analyzed. Patient background included gender, age in months at surgery, chromosomal abnormalities, prescription of prostaglandin E1 preparations before TOF repair, use of artificial materials in TOF repair, TOF surgical technique, and implementation of Blalock-Taussig shunt surgery (procedure code: 150,138,810) as palliative surgery for TOF treatment. Reinterventions were evaluated as cardiovascular surgeries or percutaneous reinterventions performed on or after the day following the index date. Medical costs were aggregated for inpatient periods associated with palliative surgery for TOF treatment, TOF repair, and reinterventions from the month of diagnosis including the index date until the end of observation.

### Statistical analysis

As this is a descriptive epidemiological study, no sample size design was performed. Reintervention-free rates were estimated using the Kaplan–Meier method. In the survival analysis, patients who did not have an event were censored at the end of their enrollment in the health insurance association or December 2022, whichever was earlier. Subgroup analyses were conducted for patient background, reintervention-free rates, and medical costs by surgical technique on the index date. Additionally, medical costs and follow-up periods for each type of reintervention were visualized using a flow chart. Furthermore, multivariable analyses using a Cox proportional hazards model were performed to explore factors associated with reintervention, with gender, age at surgery, and TOF surgical technique as explanatory variables for the period from the index date to the first reintervention. All statistical analyses were performed using SAS version 9.4.

## Results

### Patient demographics

**Fig. 1:**
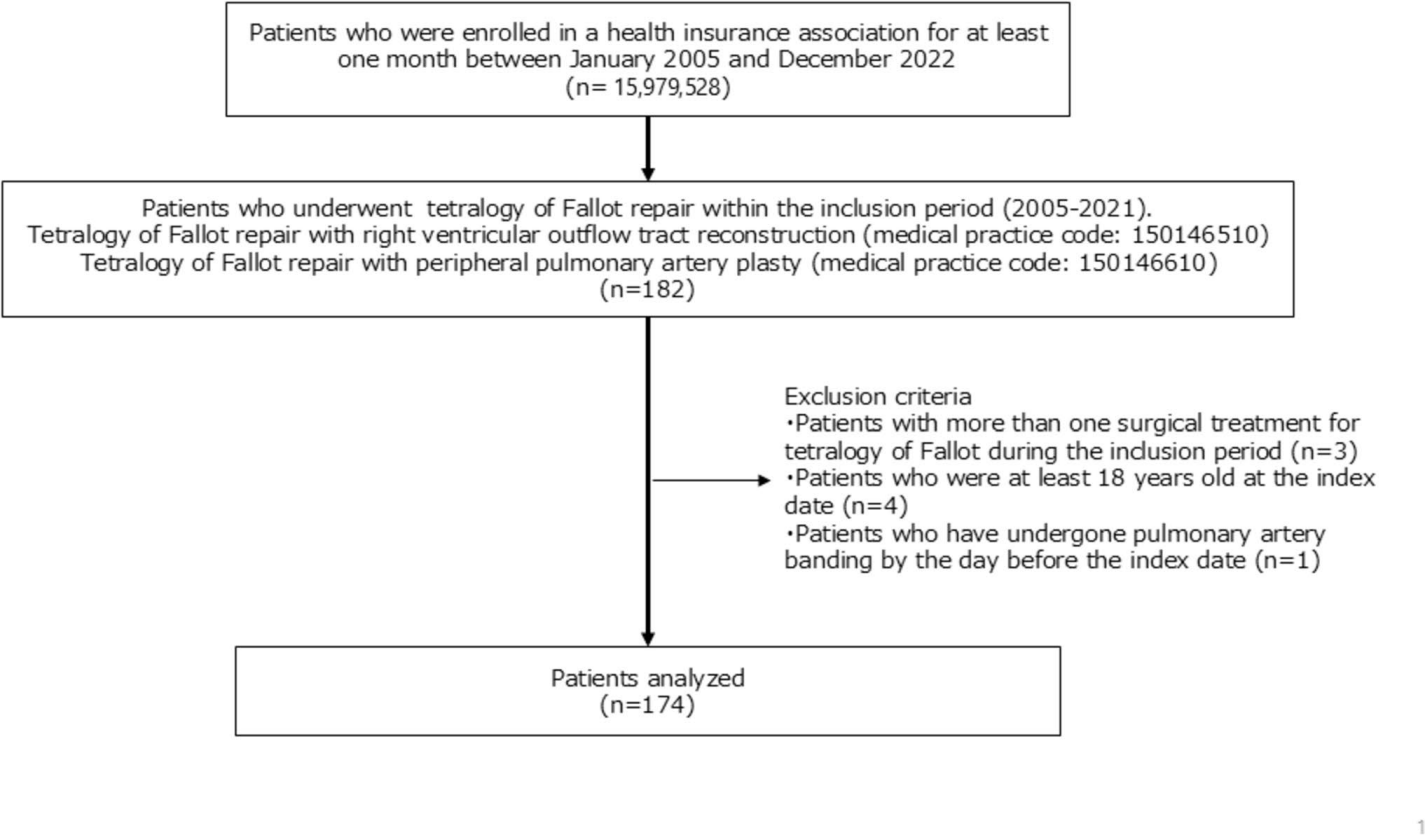
Flowchart for inclusion and exclusion criteria of patients

Of the 15,979,258 patients with confirmed insurance enrollment records during the enrollment period, 174 patients who received TOF surgical treatment and did not meet the exclusion criteria formed the analysis cohort (Fig. 1). Regarding patient background, 92 cases (52.9%) were male, and the median age at initial surgery was 13 months (10–16). Chromosomal abnormalities were observed in 27 cases (15.5%), palliative surgery (BT shunt) was performed in 39 cases (22.4%), and prostaglandin E1 preparations were prescribed to 16 cases (9.2%) before TOF repair. TOF repair (with right ventricular outflow tract reconstruction) was performed in 130 cases (74.7%), and artificial materials were used in 150 patients (86.2%) during TOF repair (Table 1).

**Table 1:**
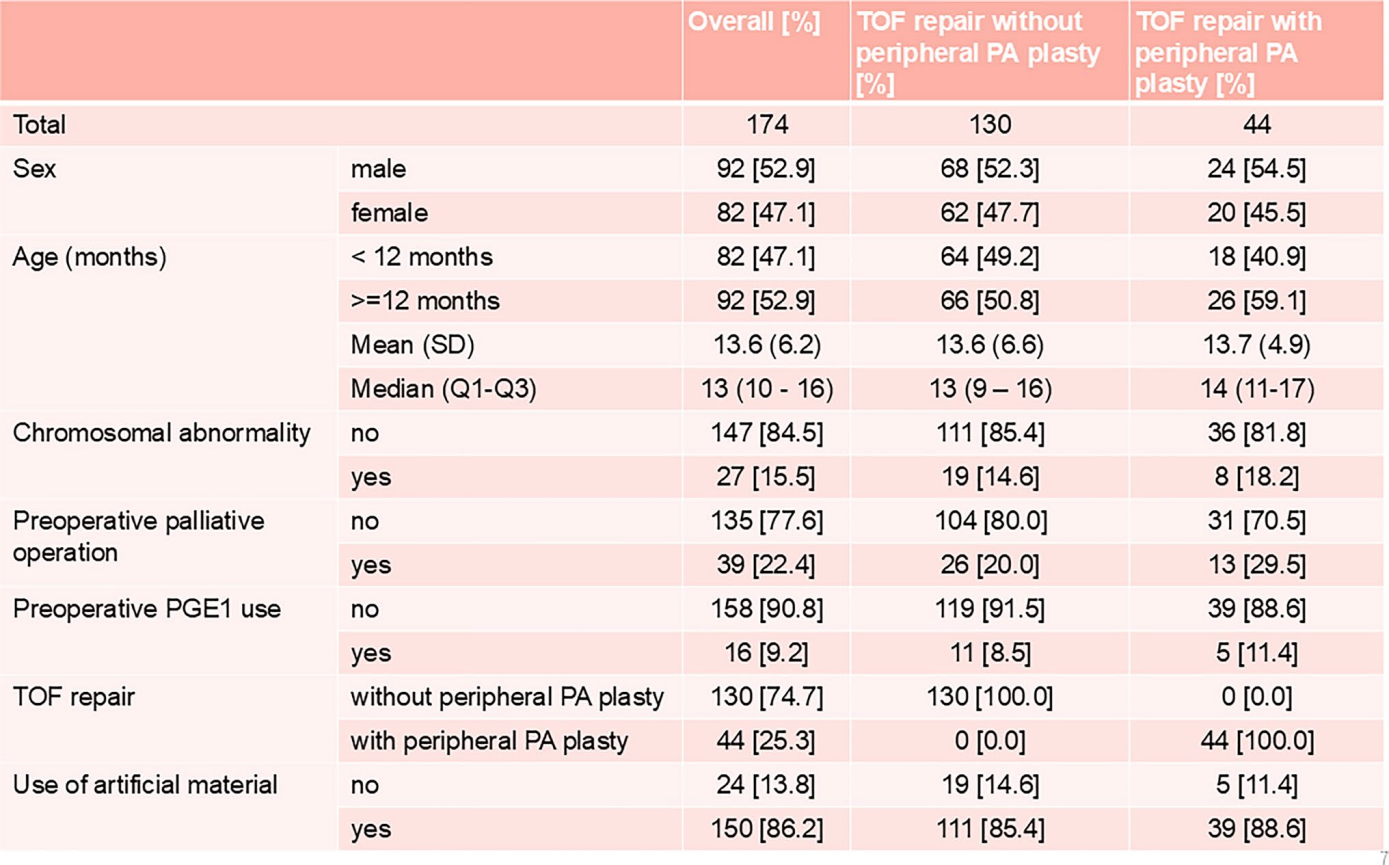
Patient demographics

### Reinterventions

The median follow-up period for the analyzed patients was 57 months (Q1-Q3: 33–99 months). A total of 34 reinterventions were performed on 23 patients (13.2%) after TOF repair. The breakdown of initial reinterventions was 14 cases (8.0%) of catheter interventions and 9 cases (5.2%) of reoperations, with a total of 23 catheter interventions and 11 reoperations performed. Catheter interventions included percutaneous pulmonary artery angioplasty (n = 21) and percutaneous pulmonary valve dilatation (n = 2). Reoperations included valve replacement surgery (n = 5), pulmonary artery plasty (n = 5), and atrial septal defect closure (n = 1).

**Fig. 2:**
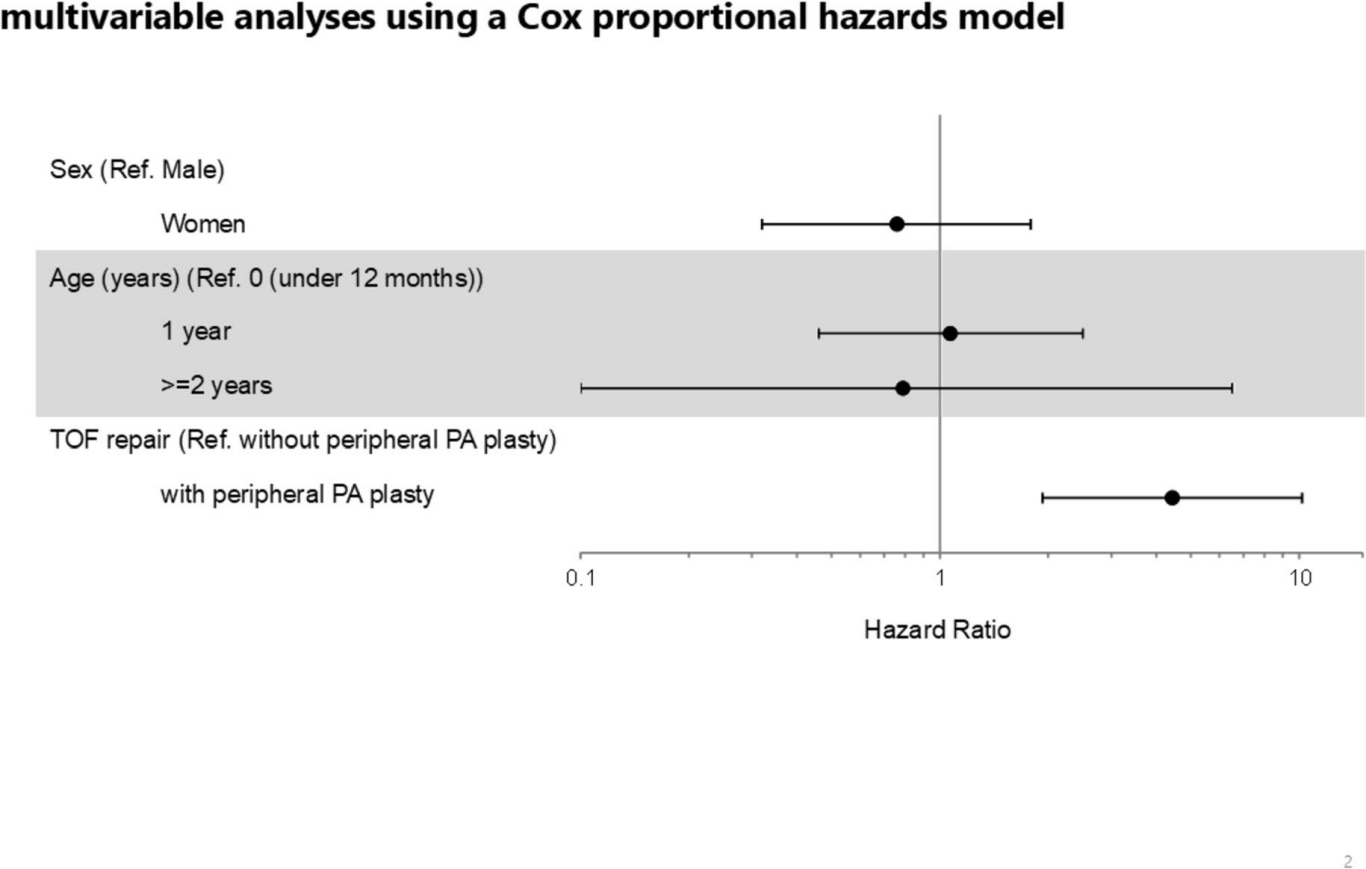
Forest plot showing the estimated hazard ratios of multivariable analyses using a Cox proportional hazards model. Circles represent the hazard ratios and the horizontal bars extends from the lower limit to the upper limit of 95% confidence interval of the estimate of the hazard ratios

In the multivariable analysis using a Cox proportional hazards model with the period from TOF repair date to reintervention as the objective variable, the hazard ratio for females was 0.76 (95% CI: 0.32–1.79, P = 0.5302) regarding gender. In the age-specific analysis, with patients under 12 months as the reference, the hazard ratios for patients aged 1 and ≧2 years were 1.07 (95% CI: 0.46–2.50, P = 0.8730) and 0.79 (95% CI: 0.10–6.50, P = 0.8270), respectively. Regarding TOF surgical technique, the hazard ratio for surgery with peripheral pulmonary artery plasty was 4.44 (95% CI: 1.93–10.19, P = 0.0004), indicating a significantly higher risk (Table 2 and Fig. 2).

**Table 2:**
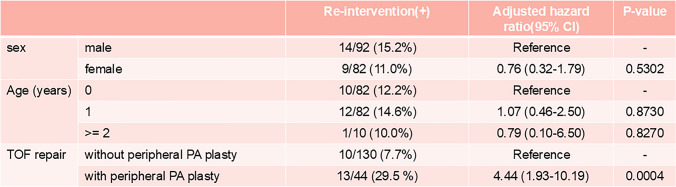
Factors related to reinterventions

**Fig. 3:**
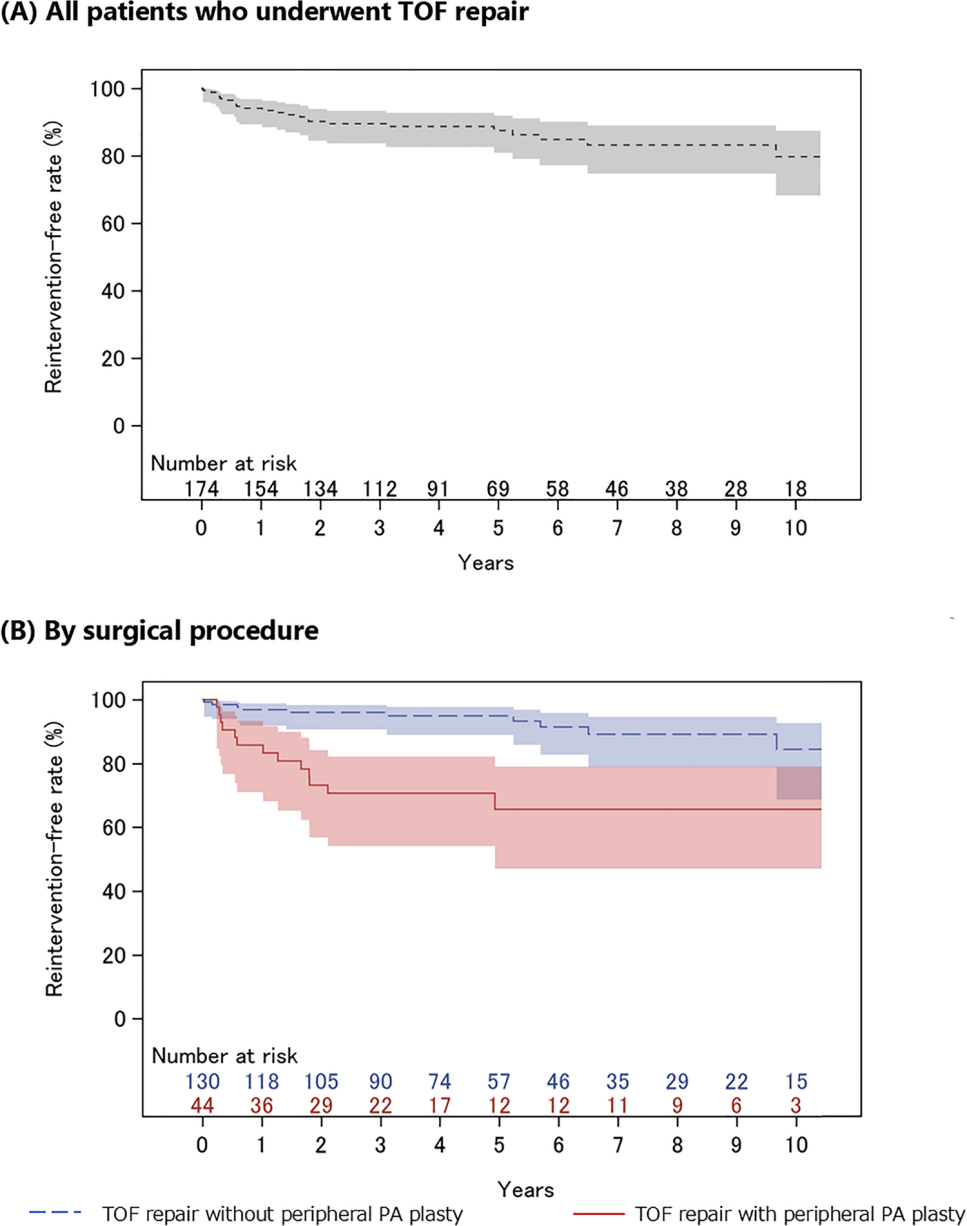
Kaplan–Meier curves for reintervention rates. **A** All patients treated with TOF repair **B** by TOF repair type. Gray solid line: all the patients who underwent TOF repair, blue dotted line; patients who underwent TOF repair without peripheral pulmonary artery plasty, red solid line; patients who underwent TOF repair with peripheral pulmonary artery plasty. The shading on the Kaplan–Meier curve indicates the 95% confidence interval for the rate of avoided reintervention

Figure 3 shows the Kaplan–Meier curves for reintervention-free rates for the entire analysis cohort and by index surgical technique. The reintervention-free rates at 1, 3, and 5 years postoperatively were 94.1%, 89.6%, and 87.5% for the entire analysis cohort; 96.9%, 96.0%, and 94.9% for patients who underwent TOF repair (with right ventricular outflow tract reconstruction); and 85.8%, 70.7%, and 65.6% for patients who underwent TOF repair (with peripheral pulmonary artery plasty).

**Fig. 4:**
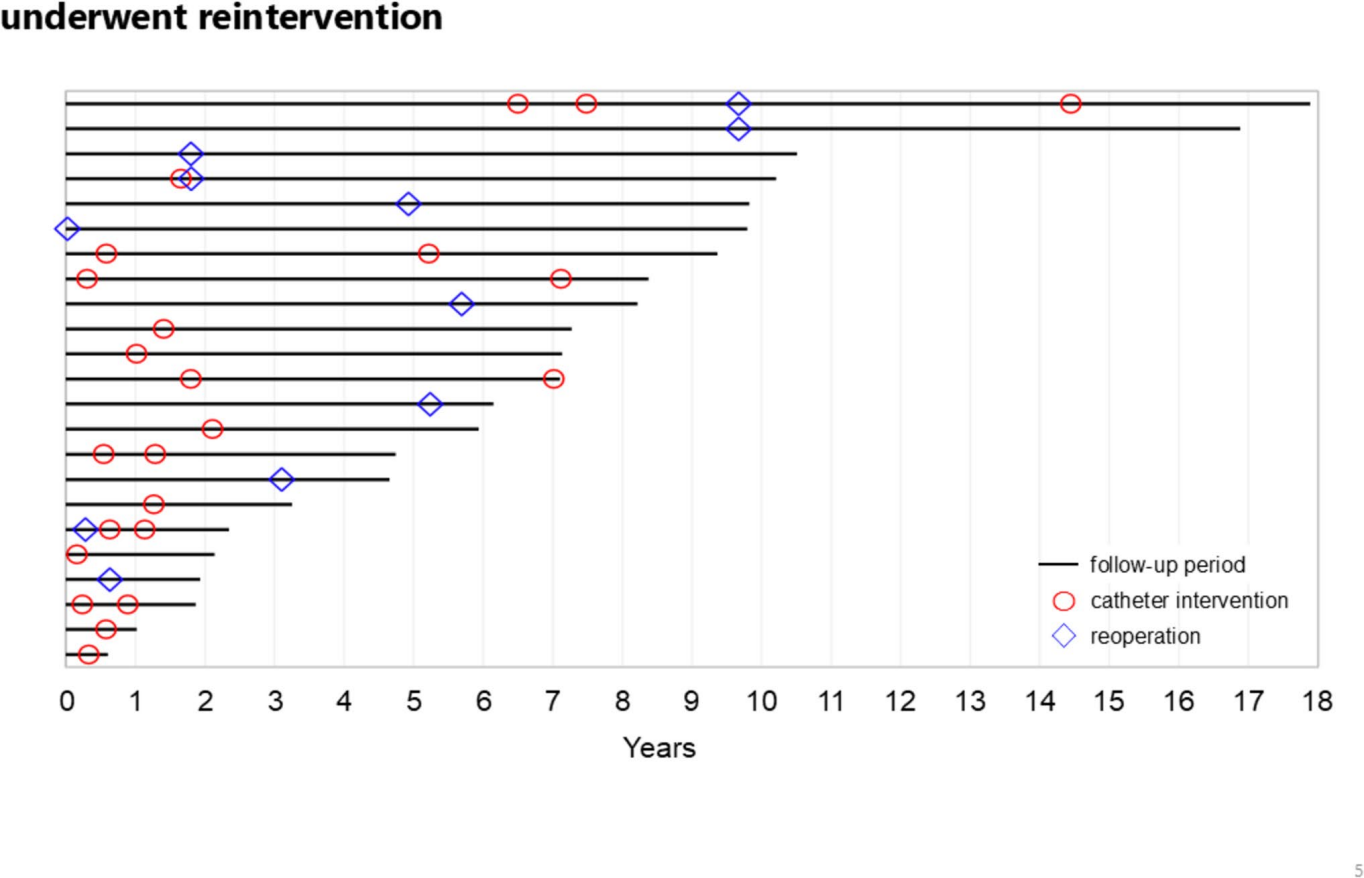
Follow-up period and timing of reintervention for each patient who underwent reintervention. Black solid line; follow-up period, red circle; percutaneous catheter intervention, blue triangle; surgical intervention. The 23 patients who underwent reintervention after TOF repair are sorted by length of follow-up

**Fig. 5:**
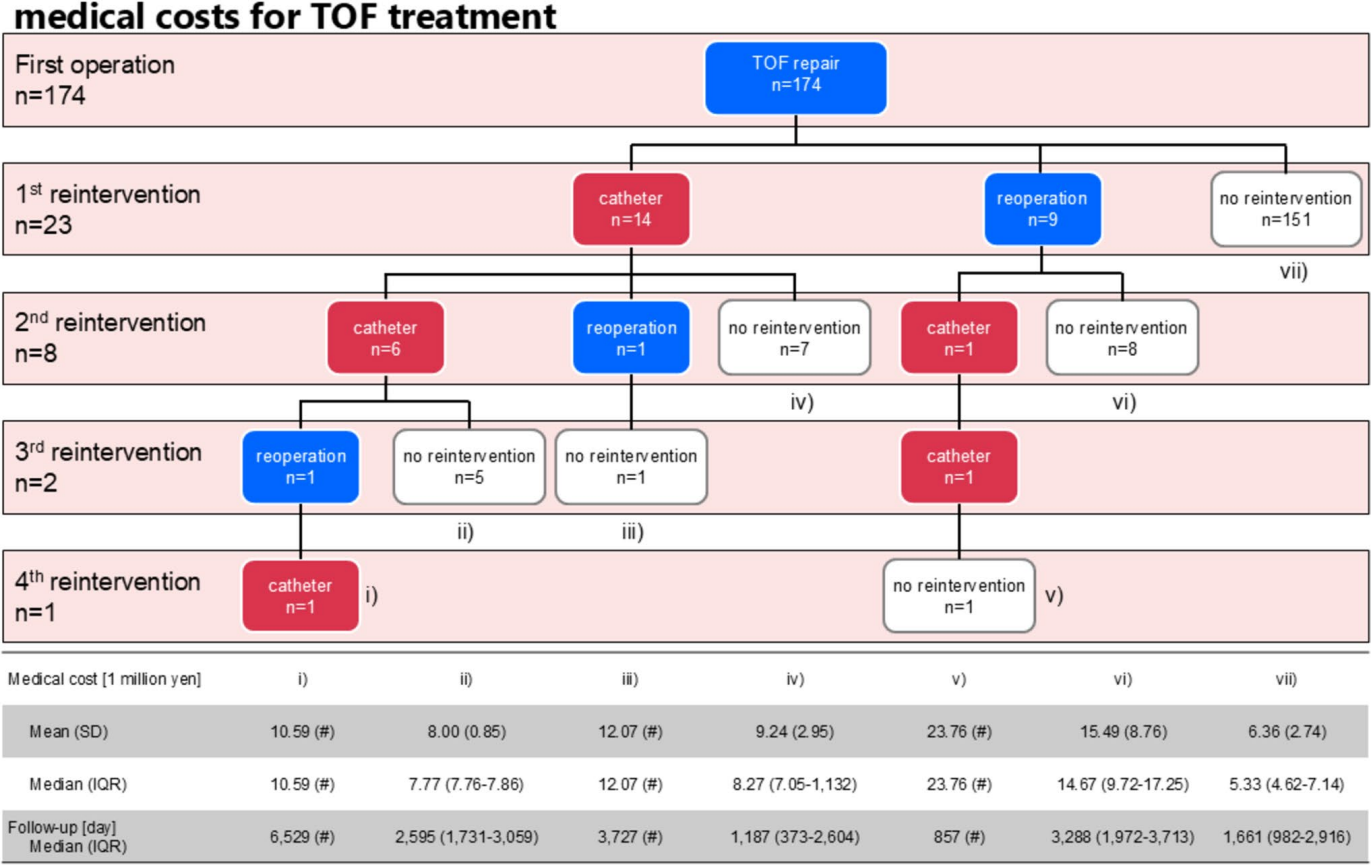
Breakdown of reinterventions, number of interventions, and medical costs for TOF treatment. #: not calculated because control group is n = 1

The types and timing of reinterventions for each case with reintervention are shown in Fig. 4. There were up to 4 reinterventions after TOF repair, with 151 patients (86.8%), 15 patients (8.6%), 6 patients (3.4%), 1 patient (0.6%), and 1 patient (0.6%) having 0, 1, 2, 3, and 4 reinterventions, respectively (Fig. 5). Age was not determined to be an associated factor for risk even when age was analyzed as a continuous variable.

Of the 174 subjects in this study, only one (0.6%) was confirmed to have died. The patient in question had undergone a palliative surgery 22 months prior to the TOF procedure and died within 1 month after the repair procedure. No reintervention was performed. There were no deaths in the reintervention group.

Medical Costs Associated with TOF Repair.

For all patients, the median (interquartile range) follow-up period was 1730 (982–3,000) days, and the median (interquartile range) medical cost for TOF treatment was ¥8.57 million (¥6.82–¥12.27 million). For patients with reinterventions (N = 23), it was ¥10.59 million (¥7.73–¥13.97 million) over 2595 (857–3576) days, and for patients without reinterventions after TOF repair (N = 151), it was ¥5.33 million (¥4.62–¥7.14 million) over 1661 (982–2,916) days. Medical costs by reintervention content and frequency are shown in Fig. 5.

## Discussion

In this analysis, we analyzed reinterventions in TOF patients using nationwide insurance claims data. The following two points were clarified from this analysis:

- Reinterventions after TOF repair were performed in about 10% of cases in 5 years, with percutaneous catheter interventions tending to be repeated multiple times.
- Factors associated with reinterventions after TOF repair included the TOF surgical technique, with postoperative outcomes worsening in cases involving peripheral pulmonary artery plasty.

By utilizing a nationwide database, this study conducted an analysis of reinterventions after TOF repair that included inter-facility differences on a national scale. Additionally, by using insurance data, it was possible to simultaneously track not only surgeries but also percutaneous catheter interventions. As the JMDC database covers only approximately 6% of the pediatric population in Japan, the generalizability of these findings to the broader national population should be interpreted with caution.

### Types and frequency of reinterventions

Reinterventions were performed 23 times as percutaneous catheter interventions and 11 times as reoperations in 23 cases (13.2%), revealing that percutaneous catheter interventions were performed more frequently. The most common reintervention procedure was percutaneous pulmonary artery angioplasty, performed 19 times, suggesting that pulmonary artery stenosis is a frequent complication after TOF repair. In patients who underwent reintervention, the median (interquartile range) period from TOF repair to the first reintervention was 15 months (5–31 months), with 70% performed within 2 years postoperatively, indicating the need for careful follow-up in the early postoperative period. 8 patients underwent two or more reinterventions, accounting for 34.8% of patients who underwent reinterventions. In 7 out of 8 cases, the initial reintervention was a percutaneous catheter intervention, indicating a tendency for percutaneous catheter interventions to be repeated multiple times, highlighting challenges in their curative nature.

### Factors associated with reinterventions

It was quantitatively demonstrated that postoperative outcomes worsened in patients who underwent TOF repair (with peripheral pulmonary artery plasty) compared to those who underwent TOF repair (with right ventricular outflow tract reconstruction). This result reflects the clinical reality that the surgical technique for patients requiring peripheral pulmonary artery plasty is more challenging, and stenosis is more likely to occur in the peripheral pulmonary arteries. It should be noted, however, that this higher risk of reintervention for peripheral pulmonary artery plasty may be influenced by confounding by indication, such as the anatomic complexity of the peripheral pulmonary artery lesion.

The follow-up period in this study covered the high-risk period from early to mid-postoperative period, as suggested by the Kaplan–Meier curve, and revealed differences in medical costs depending on the presence, type, and number of reinterventions during this period. The patient with the highest medical cost, at ¥34.43 million, underwent palliative surgery followed by TOF repair and one reoperation as a reintervention.

This study revealed that postoperative reinterventions occur at a certain frequency and increase medical costs, resulting in a high disease burden. In addition, patients with tetralogy of Fallot will most likely require further, long-term postoperative re-intervention, further increasing patient burden and lifetime medical costs. Reducing reinterventions is important from both patient burden and health economic perspectives, necessitating surgical planning that considers factors related to avoiding reinterventions. The results of this study may provide information on the need for closer monitoring after peripheral pulmonary artery plasty.

### Study limitations

Insurance claims data may lack detailed information on surgical techniques (such as valve preservation) that could influence surgical outcomes. Therefore, it is important to conduct studies in conjunction with data containing anatomical information to obtain detailed information about the clinical background and surgical techniques. Additionally, follow-up becomes impossible if the insured person leaves the health insurance society or if the contract with individual health insurance societies ends. Because the current results are based on a relatively short-term follow-up, future linkage with clinical registries may be necessary to explore long-term results.

## Conclusion

We analyzed reinterventions in TOF patients using insurance claims data, conducting simultaneous analysis of reoperations and catheter interventions, as well as trends in multiple interventions. TOF repair with peripheral pulmonary artery plasty was identified as a challenge with a high reintervention rate, suggesting the need for improved treatment methods. The results of this analysis are expected to contribute to the prediction of outcome after TOF repair.

## Data Availability

The deidentified participant data will not be shared.
